# Serial position effects differ between Alzheimer’s and vascular features in mild cognitive impairment

**DOI:** 10.18632/aging.101678

**Published:** 2018-12-12

**Authors:** Russell Jude Chander, Heidi Foo, Tingting Yong, Levinia Lim, Jayne Tan, Ming-Ching Wen, Adeline Ng, Shahul Hameed, Simon Ting, Juan Zhou, Nagaendran Kandiah

**Affiliations:** 1 Department of Neurology, National Neuroscience Institute, Singapore 308433, Singapore; 2 Department of Neurology, Singapore General Hospital, Singapore 169856, Singapore; 3 Duke-NUS Medical School, Singapore 169857, Singapore

**Keywords:** mild cognitive impairment, MRI, cerebrovascular disease, ApoE, serial position effect

## Abstract

Individuals with mild cognitive impairment (MCI) exhibit varying serial position effect (SPE) performances. The relationship between SPE performance in word list recall and clinical, genetic, and neuroimaging features of MCI requires elucidation. 119 MCI and 68 cognitively normal (CN) participants underwent cognitive assessment, apolipoprotein E (ApoE) genotyping, and volumetric MRI brain scans processed via voxel-based morphometry. A 10-word recall task was used to assess SPE performance in relation to recency and primacy recall. MCI participants were classified as having Good SPE performance (high primacy and recency, Good SPE) or Poor SPE performance (low primacy only, LP-SPE; low recency only, LR-SPE; or both low, Low SPE). Poor SPE participants had reduced grey matter (GM) volumes and increased white matter hyperintensities (WMH) volumes. Participants with LP-SPE demonstrated reduced hippocampal GM volumes and were more likely to be ApoE ε4 carriers. LR-SPE was associated with higher WMH volumes. Presence of both greater WMH volumes and ApoE ε4 resulted in Low SPE. LP-SPE MCI participants had features typical of Alzheimer’s disease. LR-SPE MCI was associated with increased WMH volumes, likely representing vascular pathology. SPE profiles are associated with distinct clinical patterns of MCI pathophysiology and could have potential as a clinical marker.

## Introduction

The serial position effect (SPE) [1,2] is a well-established psychological phenomenon of free recall. In SPE, when memorizing and recalling a list of items that exceeds the average attention span, individuals tend to preferentially recall the first and last few items over the middle items. Specifically, the ready recall of the first few items is known as the primacy effect, and the recency effect refers to the ready recall of the last few items. This effect can be seen even in individuals with normal cognition, and assumes no other method is used to facilitate memorization (e.g. via chunking or mnemonics).

SPE performance in participants with dementia has been well described in the literature. Specifically, participants with Alzheimer’s disease dementia (AD) exhibit a characteristic SPE pattern of reduced primacy effect recall with relatively preserved recency effect [3]. More recent studies have focused mainly on the primacy effect and have consistently found impaired primacy function in participants with AD [4,5]. This “AD phenotype” of SPE having a greater recency performance over primacy has been said to indicate a more passive learning approach [6], and is consistent with functional MRI studies in young adults finding an association between recognition for primacy items and increased activation of long-term memory pathways [7], pathways understood to be deficient in AD.

Unlike AD, the patterns of SPE performance in participants with mild cognitive impairment (MCI) tend to be more varied, likely due to the heterogeneous nature of cognitive deficits found in MCI. Previous work has found that decreased primacy performance distinguishes between MCI participants and controls [8] and is associated with conversion to dementia [9]. However, Martin and colleagues [10] found MCI participants to have similar SPE patterns as controls, albeit with generally poorer performance. Additionally, Moser and colleagues [4] found SPE performance for amnestic and non-amnestic MCI participants to be indistinguishable from mild AD dementia and healthy control participants respectively, with neither group exhibiting any distinct SPE pattern. It is likely that this conflicting evidence is at least partially due to the groups of MCI participants studied having significant variances in SPE performance, including MCI participants with good SPE performance and participants with poor primacy and/or recency performance. Furthermore, the inverse of AD-type SPE (i.e. participants with poor recency performance and relatively preserved primacy) is not well studied. Understanding the risk factors and pathophysiology associated with MCI participants exhibiting the various SPE profiles can help explain how SPE manifests in early neurocognitive disorders, as well as better evaluate its utility as a clinical marker for neurocognitive disease progression.

This study aims to elucidate the relationship between SPE performance and other clinical, neuropsychological, and neuroimaging features of MCI, and to identify the salient risk factors for distinguishing specific SPE profiles from control participants.

## RESULTS

### CN and MCI characteristics

**Figure 1:**
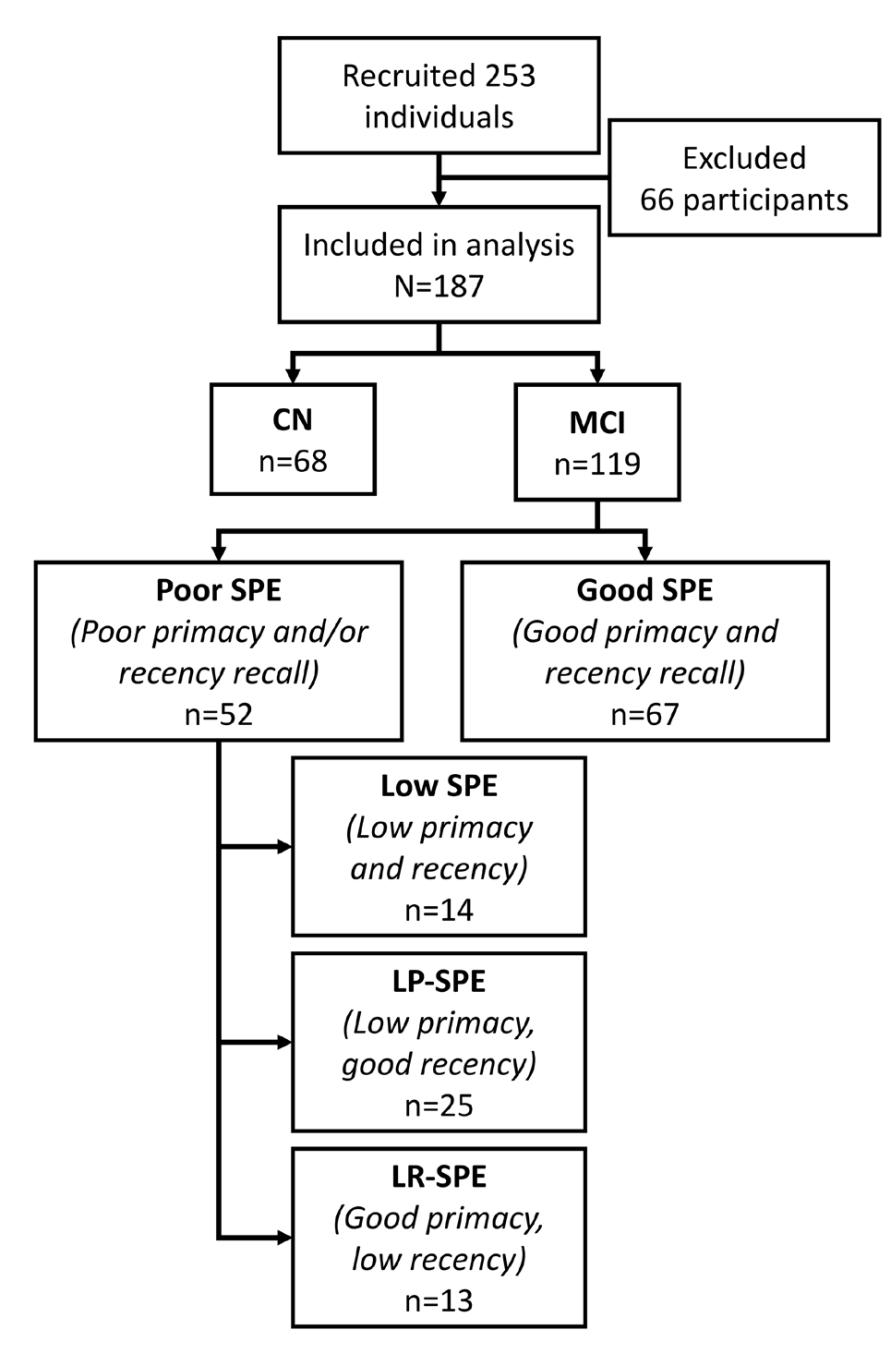
**Flowchart for study recruitment and classification into study conditions by SPE performance.** Abbreviations: AD-type: Alzheimer’s disease-type; CN: cognitively normal controls; LP-SPE: low primacy only serial position effect; LR-SPE: low recency only serial position effect; MCI: mild cognitive impairment; SPE: serial position effect.

During the study period, 253 participants were recruited and completed study procedures. From there, a further 66 participants were dropped from analysis (37 with Mini-Mental State Exam (MMSE) scores <24, 29 with MRI images failing volumetric quality control due to excessive artifacts). The current study cohort comprised of the remaining 187 participants, of whom 177 were right handed. 68 participants were classified as cognitively normal (CN) and 119 were classified as MCI (Figure 1). CN participants were significantly younger than MCI participants [mean age 62.34 (SD 6.80) vs. 67.61 (SD 7.72) years; p<0.001], were more highly educated [mean duration 13.49 (SD 3.12) vs. 11.14 (SD 3.39) years; p<0.001], and were more likely to be employed at the time [48.5% vs. 28.6%; p=0.023; remainder of participants were retired or homemakers]. CN and MCI participants did not differ in terms of gender (55.9% vs. 48.7% female; p=0.347) or race (94.1% vs. 89.9% Han Chinese; p=0.652; remainder of participants were a mix of Malay, Indian, Eurasian, and others).

**Figure 2:**
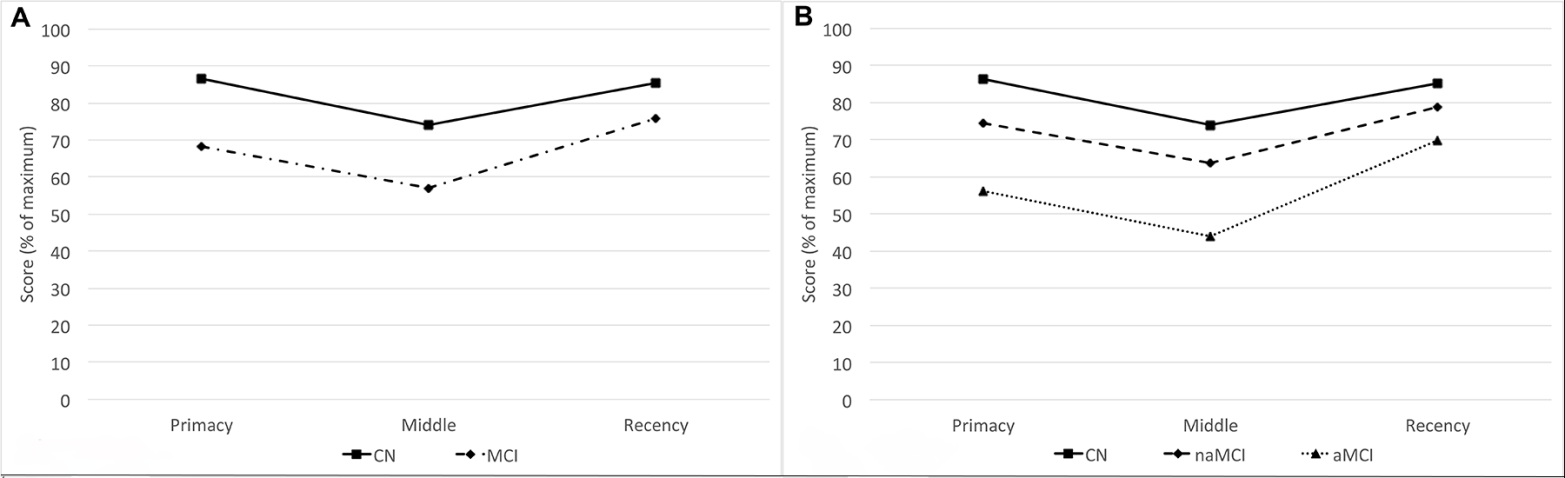
**Serial position performance for CN and MCI participants.** (**A**) Serial position performance for CN (solid line) and all MCI participants (dotted and dashed line). (**B**) Serial position performance for amnestic (dotted line) and non-amnestic (dashed line) participants. Abbreviations: aMCI: amnestic mild cognitive impairment; CN: cognitively normal controls; MCI: mild cognitive impairment; naMCI: non-amnestic mild cognitive impairment.

Of the 119 MCI participants, 40 (33.6% of MCI participants, 21.4% of cohort) were classified as amnestic MCI (aMCI). All participants performed better in the primacy and recency regions than in the middle region, and MCI participants performed worse than CN participants in all three regions while generally exhibiting the same recall patterns (Figure 2A). When considering amnestic status separately, aMCI participants have generally lower performance than CN and non-amnestic MCI (naMCI) participants while exhibiting a slightly increased recency performance over primacy performance (Figure 2B).

### SPE performance comparison

67 (56.3%) of MCI participants were classified as Good SPE, and 52 (43.7%) were classified as Poor SPE (Figure 1). Compared to CN participants, both Good SPE and Poor SPE participants were significantly older and less educated, and only the Poor SPE group had a greater proportion of Apolipoprotein E (ApoE) ε4 carriers than CN or Good SPE participants. In cognitive assessment both Good SPE and Poor SPE participants performed worse than CN in all tests at Holm-Bonferroni corrected significance levels, with Poor SPE participants generally performing poorer than Good SPE participants in all assessments except Digit Span Forward, Boston Naming Test, and the ADAS-Cog Maze (Table 1).

**Table 1:** Univariate analysis comparing CN participants with MCI participants with Good SPE and Poor SPE performance.

	**CN** **N = 68**	**MCI - Good SPE** **N = 67**	**MCI - Poor SPE** **N = 52**	**Overall p value**
Age, mean (SD), years	62.34 (6.80)	66.61 (7.82)	68.89 (7.47)	<0.001^†‡^
Race, No. (%), Chinese	64 (94.1%)	62 (92.5%)	45 (86.5%)	0.224
Gender, No. (%), female	38 (55.9%)	38 (56.7%)	20 (38.5%)	0.125
Education, mean (SD), years	13.49 (3.12)	11.42 (3.51)	10.79 (3.22)	<0.001^†‡^
Currently employed, No. (%)	33 (48.5%)	22 (32.8%)	12 (23.1%)	0.005^‡^
Diabetes mellitus, No. (%)	12 (17.7%)	16 (24.2%)	12 (23.1%)	0.616
Hypertension, No. (%)	22 (32.4%)	26 (39.4%)	23 (44.2%)	0.447
Hyperlipidemia, No. (%)	38 (55.1%)	39 (59.1%)	29 (55.8%)	0.912
History of stroke, No. (%)	3 (4.4%)	2 (3.0%)	4 (7.7%)	0.493
History of AF, No. (%)	3 (4.4%)	0 (0.0%)	2 (3.9%)	0.169
Current smoker, No. (%)	1 (1.5%)	3 (4.6%)	3 (5.8%)	0.648
Consumes alcohol, No. (%)	26 (38.2%)	14 (21.2%)	15 (28.9%)	0.036
BMI, mean (SD), kg m^-2^	23.87 (3.57)	23.38 (2.75)	23.12 (3.72)	0.545
ApoE ε4 carrier, No. (%)	10 (14.7%)	10 (14.9%)	20 (38.5%)	0.001^‡§^
MMSE score, mean (SD)	29.16 (1.00)	27.60 (1.59)	26.36 (1.61)	-
MoCA-SG score, mean (SD)	28.35 (1.30)	25.82 (2.74)	23.64 (3.25)	-
Episodic memory				
Visual Reproduction II, mean (SD)	28.15 (9.55)	23.57 (10.66)	14.98 (11.54)	<0.001^†‡§^
Word Recognition, mean (SD)*	0.51 (0.84)	1.30 (1.94)	2.98 (1.93)	<0.001^†‡§^
Attention				
Symbol search, mean (SD)	30.01 (7.83)	24.60 (6.67)	20.44 (7.00)	<0.001^†‡§^
Color Trails 1, mean (SD), sec*	50.09 (18.43)	67.29 (32.76)	82.56 (35.31)	<0.001^†‡§^
Working memory				
Digit Span Forward, mean (SD)	10.91 (2.09)	9.34 (2.49)	9.24 (2.08)	<0.001^†‡^
Digit Span Backwards, mean (SD)	9.65 (2.81)	8.10 (2.24)	7.24 (1.83)	<0.001^†‡§^
Executive function				
Color Trails 2, mean (SD), sec*	91.21 (22.05)	124.70 (49.18)	157.55 (60.51)	<0.001^†‡§^
FAB, mean (SD)	17.51 (0.68)	16.24 (2.18)	15.04 (2.19)	<0.001^†‡§^
Language				
Fruit fluency, mean (SD)	16.79 (3.47)	14.46 (3.39)	12.22 (3.89)	<0.001^†‡§^
BNT uncued, mean (SD)	26.71 (2.34)	24.36 (3.69)	23.37 (5.27)	<0.001^†‡^
Visuospatial orientation				
ADAS-Cog Maze, mean (SD), sec*	13.77 (7.08)	19.89 (13.14)	20.22 (11.51)	<0.001^†‡^
Block Design raw score, mean (SD)	42.97 (9.34)	36.62 (9.01)	31.65 (9.68)	<0.001^†‡§^

* higher values correspond to poorer outcomes; † statistically significant between CN and Good SPE participants; ‡ statistically significant between CN and Poor SPE participants; § statistically significant between Good SPE and Poor SPE participants. Abbreviations: AF: atrial fibrillation; ApoE ε4: apolipoprotein E ε4 allele; BMI: body mass index; BNT: Boston Naming Test; CN: cognitively normal; FAB: Frontal Assessment Battery; MCI: mild cognitive impairment; MMSE: Mini-Mental State Examination; MoCA-SG: Montreal Cognitive Assessment – Singapore version; SPE: serial position effect.

CN, MCI – Good SPE, and MCI – Poor SPE participants differed significantly in total grey matter (GM) volumes, as well as the bilateral occipital lobes, temporal lobes, and hippocampi. Post-hoc analysis showed that only the Poor SPE group had significantly reduced volumes compared to CN participants, and no differences were detected with the Good SPE participants. Similarly, white matter hyperintensities (WMH) volumes were significantly different, with Poor SPE participants having greater total and periventricular WMH volumes than CN participants. Global white matter (WM) volumes did not differ across the three groups (Table 2). As statistical significance was not achieved for global WM volumes, further breakdown of regional WM volumes was not explored.

**Table 2:** Univariate analysis comparing CN participants with MCI participants with Good SPE and Poor SPE performance.

	**CN** **N = 68**	**MCI - Good SPE** **N = 67**	**MCI - Poor SPE** **N = 52**	**Overall p value**
Intracranial volume, mean (SD), cm^3^	1482.46 (136.51)	1465.58 (130.72)	1502.18 (131.24)	0.323
Total GM volume, mean (SD), cm^3^	603.60 (48.91)	588.17 (49.09)	581.34 (51.53)	0.036^‡^
Frontal lobe right	77.51 (7.32)	74.75 (7.03)	74.85 (7.70)	0.054
Frontal lobe left	73.67 (6.61)	71.37 (6.87)	71.38 (7.70)	0.061
Parietal lobe right	34.20 (3.17)	33.68 (3.39)	33.62 (3.63)	0.498
Parietal lobe left	33.26 (3.01)	32.46 (3.24)	32.55 (3.51)	0.283
Occipital lobe right	25.08 (2.63)	24.16 (2.95)	23.63 (2.88)	0.030^‡^
Occipital lobe left	26.00 (2.81)	25.24 (3.12)	24.53 (3.16)	0.024^‡^
Temporal lobe right	43.83 (3.97)	42.71 (4.40)	41.68 (4.91)	0.018^‡^
Temporal lobe left	42.75 (3.97)	41.73 (4.19)	40.63 (4.55)	0.034^‡^
Hippocampus right	3.21 (0.32)	3.11 (0.29)	2.91 (0.43)	<0.001^‡^
Hippocampus left	3.31 (0.32)	3.21 (0.32)	2.99 (0.45)	<0.001^‡^
Total WM volume, mean (SD), cm^3^	475.11 (53.75)	458.60 (53.83)	458.15 (56.20)	0.092
Total WMH volume, mean (SD), cm^3^	1.69 (3.54)	2.18 (3.76)	4.46 (7.27)	<0.001^‡^
Periventricular	1.19 (2.20)	1.78 (3.03)	3.59 (5.20)	0.001^‡^

^†^ statistically significant between CN and Good SPE participants; ^‡^ statistically significant between CN and Poor SPE participants. Abbreviations: CN: cognitively normal; GM: grey matter; MCI: mild cognitive impairment; SPE: serial position effect; WM: white matter; WMH: white matter hyperintensities.

**Figure 3:**
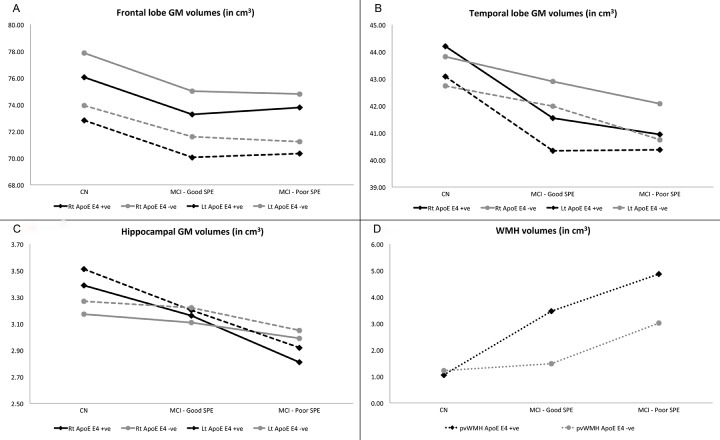
**Comparisons of volumes of neuroimaging regions of interest across SPE profiles.** Participants are also distinguished by ApoE ε4 carriers (black lines) and non-carriers (grey lines), and, where applicable, right (solid lines) and left hemispheres (dashed lines). (**A**) Frontal lobe GM volumes. (**B**) Temporal lobe GM volumes. (**C**) Hippocampal volumes. (**D**) Periventricular WMH volumes. Abbreviations: ApoE ε4: apolipoprotein ε4 allele expression; GM: grey matter; pvWMH: periventricular white matter hyperintensities; SPE: serial position effect; WMH: white matter hyperintensities.

No significant differences between study conditions or (ApoE) polymorphism were found for GM volumes in the frontal lobe (Figure 3A) or temporal lobe (Figure 3B). There were study condition x ApoE status effects found for hippocampal GM and periventricular WMH (pvWMH) volumes. For hippocampal volumes in ApoE ε4 carriers, bilateral volumes were significantly lower in Poor SPE participants (p=0.001 for right, p=0.001 for left) and left hippocampus in Good SPE participants (p=0.096 for right, p=0.023 for left) compared to CN participants. In ApoE ε4 non-carriers, bilateral hippocampal volumes were lower in Poor SPE participants compared to CN (p=0.042 for right, p=0.012 for left), and hippocampal volumes did not differ between Good SPE and CN participants (p=0.259 for right p=0.516 for left) (Figure 3C). For pvWMH, volumes were significantly greater in ApoE ε4 negative Poor SPE participants compared to CN (p=0.002), but not for Good SPE participants (p=0.947) compared to CN. In ApoE ε4 carriers, pvWMH volumes in both Good SPE and Poor SPE were greater than CN, but this did not reach statistical significance (p=0.153 for Good SPE vs. CN; p=0.089 for Poor SPE vs. CN) (Figure 3D).

### SPE profiles comparison

**Figure 4:**
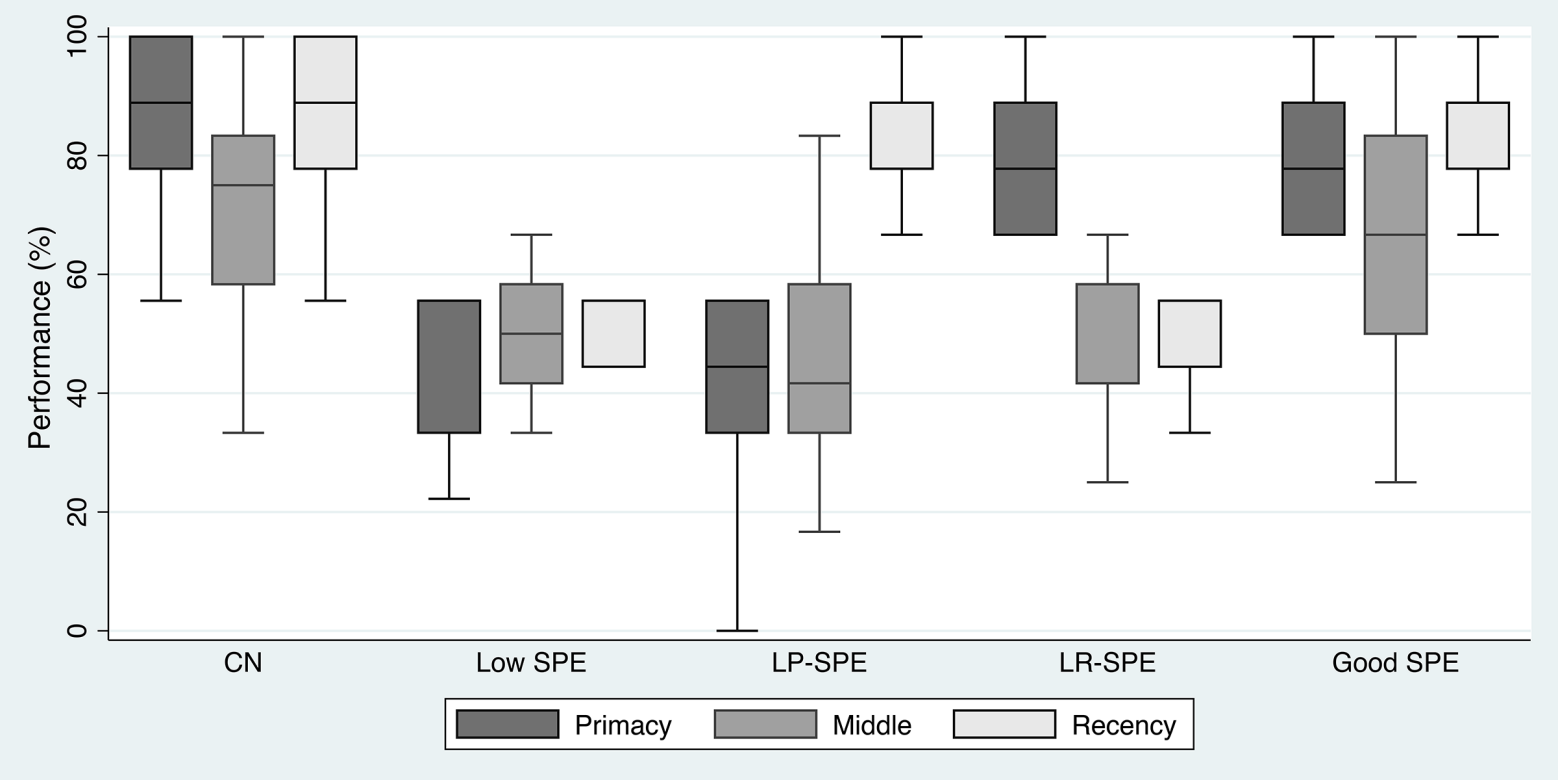
**Performance in the three SPE regions across the five study conditions.** Abbreviations: CN: cognitively normal controls; LP-SPE: low primacy-high recency SPE condition; LR-SPE: low recency-high SPE condition; SPE: serial position effect.

Of the Poor SPE participants, 14 (11.8% of all MCI participants) were classified as Low SPE, 25 (21.0%) were low primacy only SPE (LP-SPE), and 13 (10.9%) were low recency only SPE (LR-SPE) (Figure 1). Checking the performance of the three SPE regions (primacy, middle, and recency) across the five groups showed results that corresponded to their classification criteria. Although the middle region was not used to classify participants and was not the focus of this analysis, it was noted that middle region performance was similar between CN and Good SPE participants, and between Low SPE, LP-SPE, and LR-SPE. Also, even though CN participants were not selected based on their SPE performance, their performance in all three regions, including the middle region, was noted to be better than all other MCI groups (Figure 4).

In terms of cognitive performance, χ^2^ tests showed significant differences across all study conditions for all cognitive measures (Table 3). Table 4 details p values from posthoc pairwise comparisons for cognitive assessments between CN, Good SPE, LP-SPE, LR-SPE, and Low SPE participants, and statistical significance was tested at Holm-Bonferroni corrected levels. For episodic memory, CN participants universally performed better in Word Recognition than the MCI groups regardless of SPE performance. Additionally, LP-SPE participants also performed worse than CN, Good SPE, and LR-SPE participants in Visual Reproduction. LP-SPE, LR-SPE, and Low SPE participants generally performed poorer than CN participants in attention tests. Working memory performance was only significantly better in CN participants compared to Good SPE, and also compared to LP-SPE when considering Digit Span Backwards. For executive function, all MCI SPE groups performed poorer than CN participants, with LP-SPE also notably poorer than Good SPE. LP-SPE were poorer than CN in language tests, with Low SPE additionally poorer than CN in fruit fluency. Finally, LP-SPE participants were poorer than CN and Good SPE participants in Block Design, a measure of visuospatial orientation. There were very few significant differences in cognitive performances amongst the SPE profiles, the main exceptions being between Good SPE and LP-SPE. After correcting for age, education, and employment status, Low SPE participants had reduced hippocampal GM volumes and significantly greater periventricular WMH volumes compared to CN participants (Table 5).

**Table 3:** Univariate analysis of cognitive assessment performance of CN participants and MCI participants in the specific SPE profiles.

	**CN** **N = 68**	**Low SPE** **N = 14**	**LP-SPE** **N = 25**	**LR-SPE** **N = 13**	**Good SPE** **N = 67**	**K-W p value**
Episodic memory						
Visual Reproduction II, mean (SD)	28.15 (9.56)	12.57 (13.12)	14.04 (10.87)	20.92 (10.45)	23.57 (10.66)	<0.001
Word Recognition, mean (SD)*	0.51 (0.84)	4.57 (3.37)	2.44 (2.63)	2.31 (2.53)	1.30 (1.94)	<0.001
Attention						
Symbol search, mean (SD)	30.01 (7.83)	19.50 (6.94)	21.40 (7.33)	20.00 (7.34)	24.60 (6.67)	<0.001
Color Trails 1, mean (SD), sec*	50.09 (18.43)	87.91 (29.30)	80.74 (43.38)	76.84 (25.42)	67.29 (32.76)	<0.001
Working memory						
Digit Span Forward, mean (SD)	10.91 (2.09)	9.36 (2.31)	9.28 (2.09)	9.31 (1.97)	9.34 (2.49)	<0.001
Digit Span Backwards, mean (SD)	9.65 (2.81)	6.79 (1.89)	7.20 (1.94)	7.92 (1.55)	8.10 (2.24)	<0.001
Executive function						
Color Trails 2, mean (SD), sec*	91.21 (22.05)	169.45 (66.57)	163.40 (83.05)	146.11 (43.83)	126.40 (56.63)	<0.001
FAB, mean (SD)	17.51 (0.68)	15.43 (2.06)	14.68 (2.15)	15.08 (2.53)	16.24 (2.18)	<0.001
Language						
Fruit fluency, mean (SD)	16.79 (3.47)	10.93 (3.52)	12.92 (4.11)	12.69 (3.61)	14.46 (3.39)	<0.001
BNT uncued, mean (SD)	26.71 (2.34)	23.64 (4.75)	22.76 (5.46)	25.00 (5.48)	24.36 (3.69)	<0.001
Visuospatial orientation						
ADAS-Cog Maze, mean (SD), sec*	13.77 (7.07)	19.76 (7.63)	19.94 (9.59)	23.22 (17.87)	19.89 (13.14)	<0.001
Block Design raw score, mean (SD)	42.97 (9.34)	33.71 (8.65)	29.20 (9.60)	35.15 (9.86)	36.62 (9.09)	<0.001

* higher values correspond to poorer outcomes. See Table 4 for p values from posthoc pairwise comparisons. Abbreviations: BNT, Boston Naming Test; CN, cognitively normal; FAB, Frontal Assessment Battery; K-W, Kruskal-Wallis test; MCI, mild cognitive impairment; SPE, serial position effect.

**Table 4:** P values from posthoc pairwise comparisons of cognitive performance among CN participants and MCI participants in the specific SPE profiles.

	**CN vs. Good SPE**	**CN vs.** **LP-SPE**	**CN vs.** **LR-SPE**	**CN vs.** **Low SPE**	**Good SPE vs. LP-SPE**	**Good SPE vs. LR-SPE**	**Good SPE vs. Low SPE**	**LP-SPE vs. LR-SPE**	**LP-SPE vs. Low SPE**		**LR-SPE vs. Low SPE**	
Episodic memory													
Visual Reproduction II, mean (SD)	0.252	<0.001^†^	0.214	0.001^†^	<0.001^†^	0.884	0.017	0.006^†^		0.539		0.070	
Word Recognition, mean (SD)*	0.002^†^	<0.001^†^	<0.001^†^	<0.001^†^	0.032	0.134	<0.001^†^	0.467		0.512		0.107	
Attention													
Symbol search, mean (SD)	0.034	0.001^†^	0.015^†^	0.043	0.016^†^	0.094	0.223	0.976		0.503		0.994	
Color Trails 1, mean (SD), sec*	0.029	<0.001^†^	0.004^†^	<0.001^†^	0.070	0.749	0.320	0.225		0.463		0.513	
Working memory													
Digit Span Forward, mean (SD)	0.002^†^	0.021	0.096	0.283	0.943	0.939	0.786	0.754		0.413		0.832	
Digit Span Backwards, mean (SD)	0.005^†^	<0.001^†^	0.089	0.003	0.057	0.985	0.103	0.143		0.659		0.123	
Executive function													
Color Trails 2, mean (SD), sec*	0.002^†^	<0.001^†^	<0.001^†^	<0.001^†^	0.008^†^	0.597	0.159	0.114		0.253		0.573	
FAB, mean (SD)	0.002^†^	<0.001^†^	<0.001^†^	<0.001^†^	0.003^†^	0.245	0.541	0.269		0.136		0.539	
Language													
Fruit fluency, mean (SD)	0.027	0.001^†^	0.019	<0.001^†^	0.051	0.254	0.011	0.636		0.676		0.357	
BNT uncued, mean (SD)	0.003^†^	<0.001^†^	0.671	0.023	0.091	0.248	0.856	0.052		0.229		0.370	
Visuospatial orientation													
ADAS-Cog Maze, mean (SD), sec*	0.080	0.050	0.060	0.219	0.967	0.739	0.402	0.803		0.437		0.371	
Block Design raw score, mean (SD)	0.080	<0.001^†^	0.381	0.448	<0.001^†^	0.802	0.819	0.016		0.009		0.968	

* higher values correspond to poorer outcomes; ^†^ statistically significant after Holm-Bonferroni correction. Results are from multivariable linear regression correcting for age, education, and employment status. See Table 3 for raw score summary statistics. Abbreviations: BNT: Boston Naming Test; CN: cognitively normal; FAB: Frontal Assessment Battery; MCI: mild cognitive impairment; SPE: serial position effect.

**Table 5:** Neuroimaging findings across the specific SPE profiles.

	**CN** **N = 68**	**Low SPE** **N = 14**	**LP-SPE** **N = 25**	**LR-SPE** **N = 13**	**Good SPE** **N = 67**	**K-W p value**
Intracranial volume, mean (SD), cm^3^	1482.46 (136.51)	1475.29 (134.06)	1483.72 (139.05)	1561.68 (116.55)	1465.58 (130.72)	0.226
Total GM volume, mean (SD), cm^3^	603.60 (48.91)	564.85 (54.53)	586.77 (53.37)	588.20 (47.62)	588.17 (49.09)	0.058
Frontal lobe right	77.51 (7.32)	72.47 (7.88)	75.52 (8.03)	75.27 (7.16)	74.75 (7.03)	0.068
Frontal lobe left	73.67 (6.61)	69.48 (7.78)	71.50 (8.13)	72.03 (6.87)	71.37 (6.87)	0.087
Parietal lobe right	34.20 (3.17)	33.60 (3.68)	33.35 (3.58)	33.75 (3.88)	33.68 (3.39)	0.758
Parietal lobe left	33.26 (3.01)	32.23 (3.67)	32.35 (3.40)	32.83 (3.45)	32.46 (3.24)	0.451
Occipital lobe right	25.08 (2.63)	23.14 (2.62)	23.74 (2.86)	24.16 (3.29)	24.16 (2.95)	0.090
Occipital lobe left	26.00 (2.81)	23.95 (3.02)	24.63 3.29)	25.32 (3.40)	25.24 (3.12)	0.123
Temporal lobe right	43.83 (3.97)	40.31 (5.04)	42.54 (5.05)	41.84 (5.00)	42.71 (4.40)	0.050
Temporal lobe left	42.75 (3.97)	39.58 (2.24)	40.96 (4.71)	41.39 (4.05)	41.73 (4.19)	0.146
Hippocampus right	3.21 (0.32)	2.66 (0.39)	3.05 (0.43)	2.96 (0.40)	3.11 (0.29)	<0.001^†^
Hippocampus left	3.31 (0.32)	2.78 (0.45)	3.11 (0.47)	3.03 (0.37)	3.21 (0.32)	<0.001^†^
Total WM volume, mean (SD), cm^3^	475.11 (53.75)	445.49 (65.68)	454.44 (47.61)	488.61 (55.55)	458.60 (53.83)	0.044
Total WMH volume, mean (SD), cm^3^	1.69 (3.54)	7.56 (9.02)	3.32 (7.70)	4.34 (4.25)	2.18 (3.76)	<0.001
Periventricular	1.19 (2.20)	6.42 (7.39)	2.43 (4.59)	3.71 (3.80)	1.78 (3.03)	<0.001^†^

^†^ statistically significant between CN and Low SPE participants (posthoc comparison adjusted p value <0.05); ^‡^ statistically significant between CN and LP-SPE participants (posthoc comparison adjusted p value <0.05); ^§^ statistically significant between CN and LR-SPE participants (posthoc comparison adjusted p value <0.05). Overall p values were derived from K-W test, and posthoc pairwise comparisons were done via multivariable linear regression correcting for age, education, and employment status. Abbreviations: AD-type, Alzheimer’s disease type; CN, cognitively normal; GM, grey matter; K-W, Kruskal-Wallis test; LP-SPE, low primacy only serial position effect; LR-SPE, low recency only serial position effect; SPE, serial position effect; WM, white matter; WMH, white matter hyperintensities.

For the logistic regression analysis, all models started with age, secondary school completion status (equivalent to at least 10 years of formal education), total frontal, parietal, occipital, and temporal lobe GM volumes, bilateral hippocampal GM volumes, pvWMH volume, and ApoE ε4 status as candidate predictor variables before undergoing elimination, with scanner type left in as a locked covariate (Table 6). The ApoE ε4 allele, education, and pvWMH were identified to be significantly associated with the Low SPE condition (Model A). For LP-SPE participants (The AD type condition), ApoE ε4, education, and left hippocampal

**Table 6:** Logistic regression models identifying the most significant predictors for each SPE profile versus controls.

**Model A: CN vs. Low SPE** **Pseudo-R^2^ = 0.39**	**Odds Ratio**	**Std Err**	**95% CI**	**p value**
Scanner type*	1.65	1.81	0.19 – 14.20	0.649
Completed secondary school	0.02	0.03	7 x 10^-4^ – 0.55	0.021
ApoE ε4 carrier	8.63	9.38	1.02 – 72.73	0.048
pvWMH	1.43	0.19	1.10 – 1.86	0.008
				
**Model B: CN vs. LP-SPE** **Pseudo-R^2^ = 0.24**	**Odds Ratio**	**Std Err**	**95% CI**	**p value**
Scanner type*	0.83	0.51	0.25 – 2.77	0.758
Completed secondary school	0.04	0.05	0.00 – 0.40	0.006
ApoE ε4 carrier	5.29	3.50	1.45 – 19.32	0.012
Left hippocampal GM	0.18	0.16	0.03 – 0.96	0.045
				
**Model C: CN vs. LR-SPE** **Pseudo-R^2^ = 0.38**	**Odds Ratio**	**Std Err**	**95% CI**	**p value**
Scanner type*	0.70	0.52	0.16 – 3.03	0.634
Completed secondary school	0.05	0.06	0.00 – 0.55	0.015
pvWMH	1.30	0.14	1.06 – 1.61	0.014
				
**Model D: CN vs. Good SPE** **Pseudo-R^2^ = 0.12**	**Odds Ratio**	**Std Err**	**95% CI**	**p value**
Scanner type*	0.88	0.35	0.40 – 1.91	0.74
Completed secondary school	0.07	0.07	0.01 – 0.54	0.011
Age	1.06	0.03	1.00 – 1.11	0.033

* locked covariate. All models started with age, secondary school education, cortical and hippocampal GM volumes, pvWMH, and ApoE status as predictors before undergoing reverse stepwise elimination of insignificant variables. Abbreviations: AD-type: Alzheimer’s disease-type; ApoE ε4: apolipoprotein E ε4 allele; CI: confidence interval; CN: cognitively normal; DWR: delayed word recall; GM: grey matter; LP-SPE: low primacy only serial position effect; LR-SPE: low recency only serial position effect; pvWMH: periventricular white matter hyperintensities; SPE: serial position effect; Std Err: standard error.

GM volumes were significant (Model B). Only pvWMH and education were significant for the LR-SPE group (Model C), and only age and education were significant for the Good SPE group (Model D). Model fit was good for all models except Model D.

## DISCUSSION

In this analysis, we found that poor SPE performance in MCI participants was associated with poorer neuroimaging outcomes, and specifically reduced hippocampal GM volumes compared to CN amongst ApoE ε4 positive participants. MCI participants with the LP-SPE profile (the AD-type SPE profile of low primacy with high recency) had more hippocampal volume loss and greater ApoE ε4 prevalence. Participants with the LR-SPE (the inverse of the AD-type profile of high primacy with low recency) had higher WMH volumes. Participants poor in both aspects of SPE had both higher WMH volumes and higher prevalence of ApoE ε4. MCI participants with good SPE profiles mainly had age and education differences compared to controls.

These neuroimaging findings were supported in part by cognitive performance findings. LP-SPE participants were the group that more often than not performed consistently poorer than the relatively healthier groups (Good SPE and CN) in measures of episodic memory and executive function, domains known to be affected in AD. LP-SPE participants performing poorer in Visual Reproduction recall, another memory task, than LR-SPE participants further highlights the AD-like phenotype of the LP-SPE profile. LP-SPE participants did also in part perform poorer than CN in attention, language, and visuospatial orientation tasks. LR-SPE participants performed poorer than CN in attention and executive function tasks, but performance in episodic memory was not similarly poorer for all tasks. While Low SPE and Good SPE participants did generally perform poorer than CN in some tasks, especially in executive functions, results were not consistent, and many comparisons did not survive posthoc pairwise corrections.

While it has already been established that clinically diagnosed dementia [3] and MCI participants [4,10] exhibit SPE deficits, these findings show that MCI participants with “AD-like” LP-SPE might already be experiencing some level of AD-like changes in episodic memory, executive function, and hippocampal structural changes, suggestive of prodromal AD. The “inverse” LR-SPE profile, a group not well studied in prior research, have greater levels of WMH, a surrogate measure of cerebrovascular disease, as well as impairments in attention and executive function. This suggests that these participants are likely experiencing vascular cognitive impairment (VCI), where their long-term memory pathways are relatively unaffected but their cholinergic pathways have been compromised by WMH. This seems to be corroborated by how Low SPE participants have both vascular changes and risk factors for AD. Indeed, Low SPE participants seem to have markedly greater amounts of pvWMH and reduced hippocampal volumes, suggesting that these participants might be experiencing more advanced pathology than the other SPE groups.

The findings also suggest that Good SPE participants are likely not experiencing any underlying observable brain pathology, despite exhibiting some reduced cognitive performance compared to healthy controls. Although the current findings suggest age and education as the main drivers here, the relatively low pseudo-R^2^ for Model D suggests a large degree of variability not explained by the current predictors. Other factors associated with transient cognitive symptoms in the elderly, such as lifestyle factors and sleep disturbances [11], may be at play here, and further research will be needed to uncover their effects.

This study, and the preceding research, help to solidify the relationship between SPE and pathological brain changes in cognitive decline, and demonstrate that these effects can be seen even in pre-dementia participants. Given the consistency of SPE performance in relation to AD- and VCI-related pathology, SPE performance has the potential to be a viable clinical marker for these pathologies in pre-clinical neurocognitive disorders. Characterizing MCI participants by their raw recall performance as a singular number, without consideration for where the deficits lie temporally, may have the effect of making them appear to be AD-like in nature, while ignoring other potential non-AD contributions. This makes SPE profiles especially relevant for participants that score low in recall tasks, where their correctly recalled words may be loaded mainly in the primacy regions, in the recency regions, or in neither. These three scenarios would be linked to different risk profiles, and yet illicit similar total scores in immediate word recall (IWR). Furthermore, looking at IWR would circumvent some of the limitations of delayed word recall (DWR) performance across different study designs, such as the varying lengths of the latent period between the immediate and delayed tasks, and the nature of the filler task used in between, if any.

The finding of MCI participants with poor primacy performance exhibiting prodromal AD features is in agreement with prior evidence of poorer delayed primacy performance in cognitively intact individuals with smaller hippocampal volumes and increased CSF hyperphosphorylated tau levels [12], and AD-type SPE performance in asymptomatic participants with a familial history of AD [13]. The use of primacy region performance in MCI in predicting time to AD conversion has been shown to be useful alone, and enhanced when coupled with CSF amyloid investigations [14]. Increasing accuracy in identifying prodromal AD participants is becoming of increasing importance, especially in clinical trials of disease-modifying drugs, where participant selection issues are one of the many difficulties associated with the high failure rate of AD drug trials [15]. Identifying participants by their specific SPE profiles may be more accurate in distinguishing MCI participants with AD pathology from other etiologies than using primacy performance alone. It may also be feasible for the vascular-type and overall low-type SPE profiles to be used to identify VCI or mixed etiology participants for clinical trials focusing on those patient groups, although further work may be needed to properly elucidate the mechanisms behind the non-AD SPE profiles.

This study is novel in its use of distinct SPE profiles to characterize MCI participants more informatively, rather than studying individual aspects of SPE alone. Despite this, certain limitations need to be acknowledged. This includes the pseudorandom nature of the Alzheimer’s Disease Assessment Scale – Cognitive (ADAS-Cog) IWR task used in this design; while this was done to avoid the effects of word association or mnemonics in aiding recall, it also meant that the serial position of the subsequent DWR words were unrelated to IWR and had no inherent significance beyond the raw score. Thus, SPE performance in DWR could not be simultaneously studied in this design.

In conclusion, we find that specific SPE profiles in MCI are suggestive of underlying AD or vascular pathology, and demonstrate it to be a useful clinical marker that is consistent and easy to administer. Further studies will be needed to demonstrate these effects in larger cohorts, and to observe the associations of these SPE profiles with amyloid or tau imaging, CSF investigations, and functional connectivity via fMRI studies.

## MATERIALS AND METHODS

### Subject recruitment

Participants were from a cross-sectional research study carried out at the National Neuroscience Institute, Singapore and Duke-NUS Medical School, Singapore that recruited individuals with MCI and healthy controls between July 2013 and March 2016. All participants were between 50 and 90 years of age at time of recruitment, were literate, had no major psychiatric or neurological comorbidities, and had no contraindications to high-field MRI. MCI participants were individuals that presented to a tertiary memory clinic for cognitive complaints, and were classified via clinical assessment using Petersen’s criteria [16] corroborated by scores in the Mini-Mental State Examination (MMSE) [17] and the Singaporean version of the Montreal Cognitive Assessment (MoCA-SG) [18]. Participants were dropped from this analysis if they had a Clinical Dementia Rating (CDR) [19] Global score greater than 0.5, MMSE score less than 24, met criteria for dementia, or were flagged for significant depressive symptoms on the 15-point Geriatric Depression Scale (GDS) [20]. Cognitively normal (CN) controls were recruited from the community via outreach programs for dementia awareness and snowball recruitment. CN participants had a CDR global score of 0, MMSE score of 27 or greater, were functionally independent, and did not meet criteria for MCI, dementia, or any other neurological or psychiatric disorder. Participants provided voluntary informed consent prior to collection of any research data.

### Cognitive and SPE assessment

All participants attended a clinical interview session where demographic data, clinical measurements, and past medical history data was noted. Cognition was assessed with the MMSE [17], MoCA-SG [18], and the Alzheimer’s Disease Assessment Scale – Cognitive (ADAS-Cog) battery [21]. Furthermore, participants underwent a comprehensive cognitive assessment battery designed to assess the following cognitive subdomains: episodic memory via the fourth version of the Wechsler Memory Scale (WMS-IV) Visual Reproduction II subtest [22] and the ADAS-Cog Word Recognition test [21], attention via the fourth version of the Wechsler Adult Intelligence Scale (WAIS-IV) Symbol Search subtest [23] and the Color Trails Test (CTT) 1 subtest [24]; working memory via the WAIS-IV Digit Span Forward and Backward subtests [23], executive function via the CTT 2 subtest [24] and the Frontal Assessment Battery [25]; language via fruit semantic fluency and the Hong Kong version of the Boston Naming Test (BNT) [26]; and visuospatial orientation via the ADAS-Cog Maze subtest [21] and the WAIS-IV Block Design subtest [23].

SPE performance was assessed using the 10-word immediate word recall (IWR) task in the ADAS-Cog [21]. Participants were shown a list of 10 sequential words on flash cards at approximately two seconds per word. The words were one or two syllables long and were relatively common concrete nouns. Participants were told to read each word out loud and to memorize them. At the end of the exposure, participants were asked to repeat as many words as they can regardless of order, taking up to a maximum of two minutes. The trial exposure and recall challenge was then repeated two more times, each using the same 10 words in a pseudorandomized order. After approximately 5 minutes, during which a filler task is conducted (in this case, the ADAS-Cog Instructions and Constructional Praxis tasks), a delayed word recall (DWR) task is carried out. During DWR, participants are challenged to repeat as many of the 10 words from the immediate word with no repeat exposure prior to challenge.

In each IWR trial, the primacy region was defined as the first three words, and the recency region was defined as the last three words. The remaining four words were labeled as the middle region. A primacy score would then be generated using the total number of correctly recalled words in the primacy regions of all three trials, out a total possible score of 9. A score of <6 (<2 out of 3 words for each trial on average) denoted poor primacy performance. Recency scores, and classifications for poor recency performance, were similarly derived using the nine recency region words. These criteria were used to classify MCI participants into the various SPE groups as described below. Separately, MCI participants also were classified as amnestic (aMCI) if the standardized z-score of either episodic memory subtest (Visual Reproduction or Word Recognition) was >1.5 standard deviations below the mean, while other MCI participants were classified as non-amnestic (naMCI).

### ApoE genotyping

Genomic DNA was extracted from peripheral blood samples using the QIAamp DNA Blood Maxi Kit (Qiagen GmbH, Hilden, Germany) according to manufacturer specifications. The TaqMan SNP genotyping assay [rs429358 (ABI assay ID: C_3084793_20) and rs7412 (ABI assay ID: C_904973_10)] on the ABI 7900HT PCR system (Applied Biosystems, Foster City, CA) was used for apolipoprotein E (ApoE) genotyping [27]. Participants with at least one copy of the ApoE ε4 allele, a risk factor for AD [28], were classified as ApoE ε4 carriers, while other participants were classified as non-carriers.

### MRI protocols and volumetric analysis

Participants underwent MRI in a 3T Siemens Tim Trio (Siemens, Erlangen, Germany; for participants recruited up until July 2014) or 3T Siemens Prisma (Siemens, Erlangen, Germany; for participants recruited after July 2014) whole body MR system. 3D volumetric scans were obtained using a T1-weighted magnetization-prepared rapid gradient-echo (MPRAGE) sequence [repetition time (TR)=2300ms, echo time (TE)=2.98ms, matrix=192x256×256, flip angle=80, 180 slices, 1.0mm isotropic voxels] as per the Alzheimer’s Disease Neuroimaging Initiative (ADNI) protocols (http://adni.loni.usc.edu/). Voxels of white matter hyperintensities (WMH) were obtained using T2-weighted fluid attenuated inversion recovery (FLAIR) sequences (TR=8000ms, TE=336.86ms, matrix=256 x 256, slice thickness=2mm, flip angle=900, 180 slices), using the corresponding MPRAGE sequence as a template.

Voxel-based morphometry (VBM) was done using the Computational Anatomy Toolbox (CAT12) package for the Statistical Parametric Mapping 12 (SPM12) software (http://www.fil.ion.ucl.ac.uk/spm) in MATLAB. Volumetric MPRAGE sequences were converted from DICOM to 3D NIfTI format and manually oriented to be within the standard Montreal Neurological Institute (MNI) template space. Images were then segmented into grey matter (GM), white matter (WM), and cerebrospinal fluid (CSF) maps using a unified segmentation pipeline [29] that includes affine regularization to the International Consortium for Brain Mapping (ICBM) space template for East Asian brains, bias corrections, and affine and non-linear modulated normalization. The generated GM and WM maps were then smoothed (8mm full width at half maximum) in SPM12. CAT12 was used to estimate the total intracranial volume (TIV) for each participant, and the smoothed GM and WM maps were used to generate global volumes of GM and WM, and also regional volumes based on regions of interest (ROIs) defined using the Wake Forest University PickAtlas v3.0 software toolbox [30].

Volumetric analysis of WMH was done in SPM12 using an existing workflow [31]. Briefly, FLAIR sequences were converted into 3D SPM/Analyze data format, and coregistered with their corresponding MNI-normalized MPRAGE sequences. WMH maps were segmented from FLAIR sequences based on voxels identified as having an intensity of at least 1.4 times compared to the modal intensity of the surrounding white matter. ROIs extending 10mm from the lateral ventricles were used to define periventricular WMH (pvWMH).

### Statistical analysis

MCI participants were classified into various groups according to their SPE performance, specifically based on if their recency and primacy recall were defined as good or poor as described above (see ‘Cognitive and SPE assessment’ subsection in Methods). MCI participants were classified as having Good SPE if they had high performance (≥6 out of 9 items correctly recalled) in both primacy and recency regions, or Poor SPE if they had poor performance (<6 out of 9 items correctly recalled) in either primacy and/or recency regions. Poor SPE participants were also further divided into low primacy and recency (Low SPE), low primacy and high recency (LP-SPE, the typical AD pattern of SPE), and high primacy and low recency (LR-SPE, the “inverse” phenotype to the AD pattern). Figure 1 details the recruitment breakdown and subclassification workflow for SPE subtypes. Subject groups were compared for differences in demographic profiles, clinical and vascular risk factors, cognitive performance, and neuroimaging variables including global and regional GM, WM, and WMH, correcting for age, education, and employment status. These volumes were also compared between CN, Good SPE, and Poor SPE participants separately for ApoE ε4 carriers and non-carriers. Logistic regression models were run to identify specific neuroimaging and risk factor features significantly associated with Low SPE (Model A), LP-SPE (Model B), LR-SPE (Model C), and Good SPE (Model D) compared to CN participants, while correcting for confounding factors, inclusive of scanner type.

All statistical analyses were done using Stata version 14 (StataCorp, College Station, TX, USA). Variables were tested for normality of distribution by appraising skewness and kurtosis, and via Shapiro-Wilk test. Univariate analysis was conducted via analysis of variance or Kruskal-Wallis test for continuous parametric and nonparametric variables respectively, and χ^2^ test for categorical variables. Significance tests were two-tailed, and level of significance for univariate analysis and reverse elimination in regression models was set at p<0.05. Post-hoc pairwise comparisons were conducted using Mann-U-Whitney test and χ^2^ test for continuous and categorical variables respectively using a significance level of p<0.05, adjusted via Holm-Bonferroni corrections to control for familywise error rate. All study procedures were carried out in accordance with institutional guidelines, and in compliance with the International Conference on Harmonization-Good Clinical Practice (ICH/GCP) and the Singapore Good Clinical Practice (SG-GCP) guidelines. The study was approved by the SingHealth Centralized Institutional Review Board (reference number 2013/267/A, dated 4^th^ April 2013).
